# RN765C, a low affinity EGFR antibody drug conjugate with potent anti-tumor activity in preclinical solid tumor models

**DOI:** 10.18632/oncotarget.26002

**Published:** 2018-09-11

**Authors:** Oi Kwan Wong, Thomas-Toan Tran, Wei-Hsien Ho, Meritxell Galindo Casas, Melinda Au, Marjorie Bateman, Kevin C. Lindquist, Arvind Rajpal, David L. Shelton, Pavel Strop, Shu-Hui Liu

**Affiliations:** ^1^ Oncology R&D, Cancer Immunology Discovery Unit, Pfizer Inc., South San Francisco, CA, USA; ^2^ Bristol-Myers Squibb, Redwood City, CA, USA; ^3^ NGM Biopharmaceuticals, South San Francisco, CA, USA; ^4^ Alector Inc., South San Francisco, CA, USA; ^5^ acib GmbH Graz, Graz, Austria; ^6^ Allogene Therapeutics, South San Francisco, CA, USA; ^7^ Abmart Inc., Redwood City, CA, USA

**Keywords:** EGFR, antibody-drug conjugate, site-specific conjugation, non-small cell lung cancer, target therapy

## Abstract

Epidermal growth factor receptor (EGFR) is a clinically validated target and often overexpressed in some solid tumors. Both EGFR tyrosine kinase inhibitors and ligand-blocking antibodies have been approved for treatment of NSCLC, head and neck cancers and colorectal cancers. However, clinical response is limited and often accompanied by significant toxicities due to normal tissue expression. To improve the effectiveness of targeting EGFR while minimizing the toxicities on normal tissues, we developed a low-affinity anti-EGFR antibody drug conjugate (ADC), RN765C. Potent *in vitro* cytotoxicity of RN765C, with nanomolar to subnanomolar EC50, was observed on a panel of cancer cell lines expressing moderate to high level of EGFR. In contrast, RN765C was less effective in killing normal human keratinocytes, presumably due to its lower receptor expression. Mechanistically, RN765C has multiple modes of action: inducing payload mediated mitotic arrest and cell death, blocking EGFR pathway signal and mediating antibody dependent cell cytotoxicity. In preclinical studies, a single dose of RN765C at 1.5-3 mg/kg was generally sufficient to induce tumor regression in multiple cell line and patient-derived xenograft models, including those that are resistant to EGFR-directed tyrosine kinase inhibitors. Our data support further investigation of RN765C in the clinic to treat EGFR expressing solid tumors.

## INTRODUCTION

EGFR is a member of the ErbB family of type I receptor tyrosine kinases. It plays essential roles in the development and normal physiology of epithelial cells, including cell proliferation, growth, differentiation, migration and inhibition of apoptosis [1]. Dysregulation of EGFR signaling through overexpression of the receptor or hyper-activation of its kinase activity is a common theme in several solid tumors including non-small cell lung cancer (NSCLC), head and neck cancer (HNSCC), colorectal cancer, breast cancer and glioblastoma multiforme. A clinically proven strategy to treat EGFR driven tumors is to block the EGFR pathway signaling. To this end, both monoclonal ligand blocking antibodies such as cetuximab and panitumumab as well as EGFR tyrosine kinase inhibitors (TKIs; erlotinib, gefitinib, lapatinib, afatinib, osimertinib) have been approved [2]. Although clinical response was initially observed, most patients developed resistance to the therapies [2-4]. In addition, treatment related moderate to severe cutaneous and gastrointestinal toxicities were often observed [5]. Thus, more effective treatment of EGFR positive tumors is needed.

Because EGFR is widely overexpressed in many types of solid tumor, we aimed to develop a more broadly applicable therapy that does not solely rely on blocking of the EGFR signaling. Antibody drug conjugates (ADCs) exploit the binding specificity of monoclonal antibodies as a mechanism for selective delivery of cytotoxic agents to kill tumor cells. ADC therapies have been approved for both solid and hematologic malignancies [6, 7] and represent an attractive modality to target EGFR positive tumors. In this paper, we described a novel EGFR ADC, RN765C, comprised of a humanized anti-EGFR hIgG1 antibody, conjugated with the AcLys-VC-PABC-PF-06380101 linker payload at the C-terminus of the antibody light chain via an enzymatic process as described previously [8, 9]. This conjugate contains a valine-citruline cleavable linker and PF-06380101 [10], which is a potent anti-mitotic agent that inhibits tubulin polymerization, leading to mitotic arrest and cell death. The antibody component of RN765C is designed to have low affinity such that it only transiently interacts with its target on normal tissue where the EGFR density is low in general. In contrast, on EGFR overexpressing tumor cells, the high receptor density allows RN765C to establish stable bivalent binding leading to efficient ADC internalization and release of the payload to kill tumor cells. Indeed, RN765C showed potent *in vitro* cell killing activity in EGFR high tumor cell lines, even in those that were non-responsive to cetuximab. In contrast, RN765C was less effective in killing normal human epidermal keratinocytes *in vitro*. *In vivo* testing of RN765C in cell line and patient-derived xenografts (PDX) models representing several solid tumor types confirmed robust activity often inducing tumor regression with a single dose. Importantly, RN765C is active towards tumors harboring mutations that are known to drive resistance to EGFR monoclonal antibody therapies (e.g. KRAS mutation) and EGFR TKIs (e.g. EGFR T790M mutation).

## RESULTS

### Generation of affinity variants of EGFR antibodies

**Figure 1:**
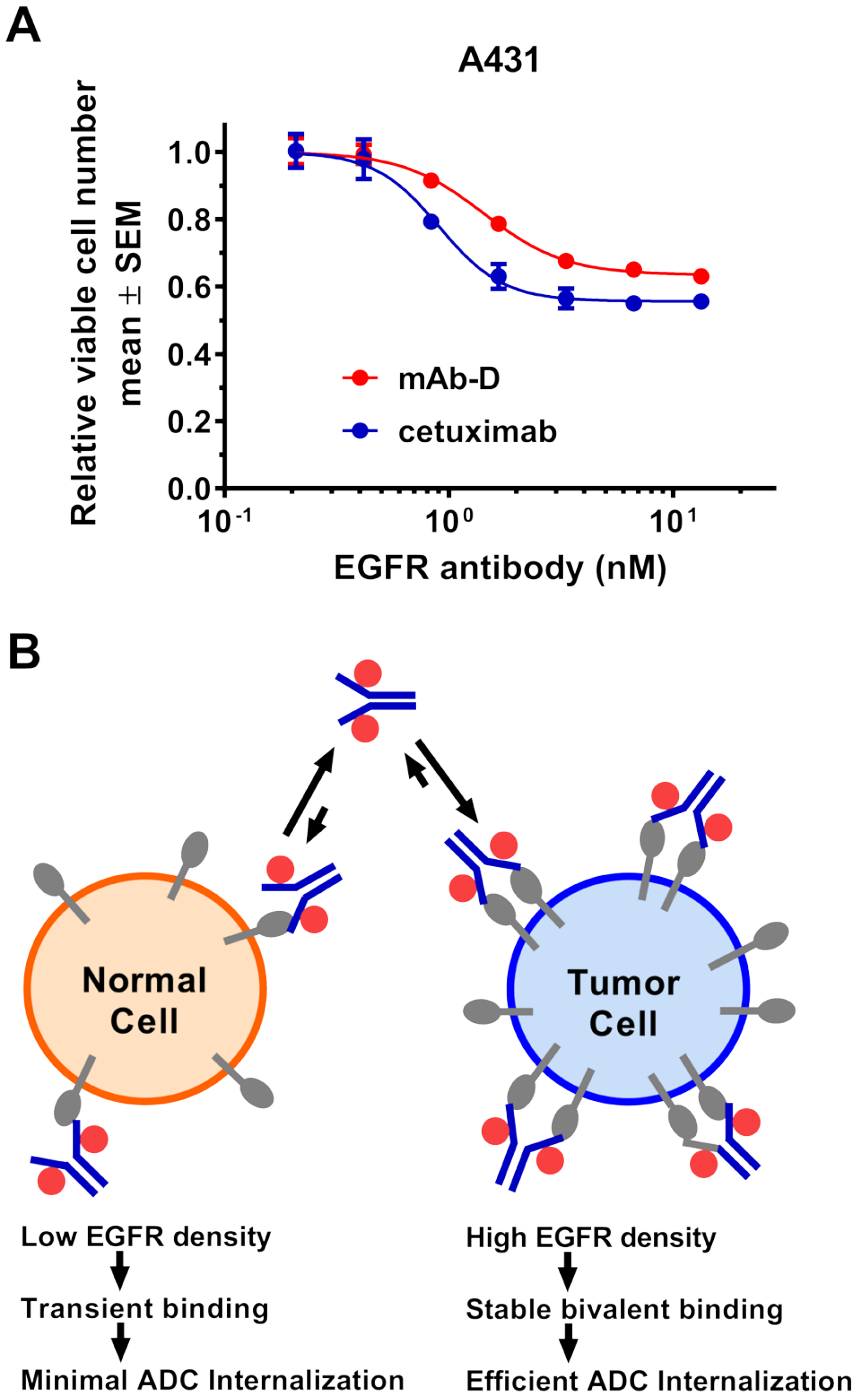
Strategy to develop an effective EGFR ADC (**A)** EGFR antibodies only partially inhibit growth of A431 cells. The ability of mAb-D (our in-house monoclonal anti-EGFR antibody) and cetuximab to inhibit A431 cell growth was performed over a treatment time of 5 days in DMEM medium + 5% FBS. Viable cell number remaining in the cultures was assessed using CellTiter-Glo^®^ and normalized to the value from wells treated with isotype control antibody. Each treatment was done in triplicate. (**B)** Schematic representation to illustrate the differential binding and internalization of low affinity EGFR-ADC on normal cells versus tumor cells that express higher level of the receptors.

Inhibition of tumor growth by blocking ligand binding to EGFR is one of the mechanisms of action of approved EGFR targeting antibodies. While pathway inhibition can slow growth of EGFR-dependent tumors, it rarely completely eliminates them. As shown in Figure 1A, both cetuximab and our in-house monoclonal mouse anti-EGFR antibody mAb-D only moderately slow down growth of A431 cells *in vitro*, presumably due to ligand inhibition, and none of them achieve more than 50% cell killing. To improve anti-tumor efficacy without exacerbating toxicity to normal tissue, we aimed to develop a low affinity anti-EGFR ADC. EGFR is frequently overexpressed in many solid tumors whereas its expression level is relatively lower in normal tissue. Taking advantage of this differential expression, we reason that in tumor cells where the EGFR expression is high, a low affinity EGFR ADC can achieve stable bivalent binding to the receptors, leading to efficient ADC internalization and killing of tumor cells. In contrast, most normal cells express EGFR at lower density and it stands to reason that a low affinity EGFR ADC would only bind to one receptor transiently most of the time. This monovalent interaction is short-lived and therefore ADC internalization is limited. Consequently, normal cells should be less susceptible to EGFR ADC mediated killing (Figure 1B).

To select EGFR antibody with an optimal affinity for generation of ADC, the mouse antibody mAb-D was humanized to generate EGFR.Ab-M. High and low affinity variants of EGFR.Ab-M were generated by screening a library containing single amino acid mutants of the light-chain variable domain, resulting in discovery of antibodies with human EGFR affinities of 16.1 nM (EGFR.Ab-H), 196 nM (EGFR.Ab-M), and 338 nM (EGFR.Ab-L) antibodies (Table 1). The affinity differences between these three variants are mainly driven by their dissociation rates and the binding kinetics of these variants are very similar for human and cynomolgus EGFR proteins. Since these EGFR antibodies only differ from each other by one amino acid residue in the light-chain variable domain, we do not expect that it will significantly change the binding epitope on the receptor. Thus, these affinity variants allow us to investigate the effect of affinity on antibody uptake in subsequent assay.

**Table 1:** Binding kinetics of EGFR affinity variant antibodies to human and cynomolgus EGFR proteins at 37^o^C

EGFR antibodies	human EGFR				cyno EGFR			
	*k*_a_ (1/Ms)	*k*_d_ (1/s)	*K*_D_ (nM)	t_1/2_ (s)	*k*_a_ (1/Ms)	*k*_d_ (1/s)	*K*_D_ (nM)	t_1/2_ (s)
EGFR.Ab-H	6.29E+05	1.01E-02	16.1	68.6	6.10E+05	8.65E-03	14.2	80.1
EGFR.Ab-M	5.35E+05	1.05E-01	196	6.60	4.90E+05	8.77E-02	179	7.90
EGFR.Ab-L	6.21E+05	2.10E-01	338	3.30	5.50E+05	1.87E-01	341	3.71

Affinity of EGFR antibodies to recombinant monomeric 8xHis-tagged extracellular domain of human EGFR and cynomolgus EGFR protein was determined by surface plasmon resonance.

### Low affinity EGFR antibody was efficiently taken up by cancer cells but not normal cells

**Figure 2:**
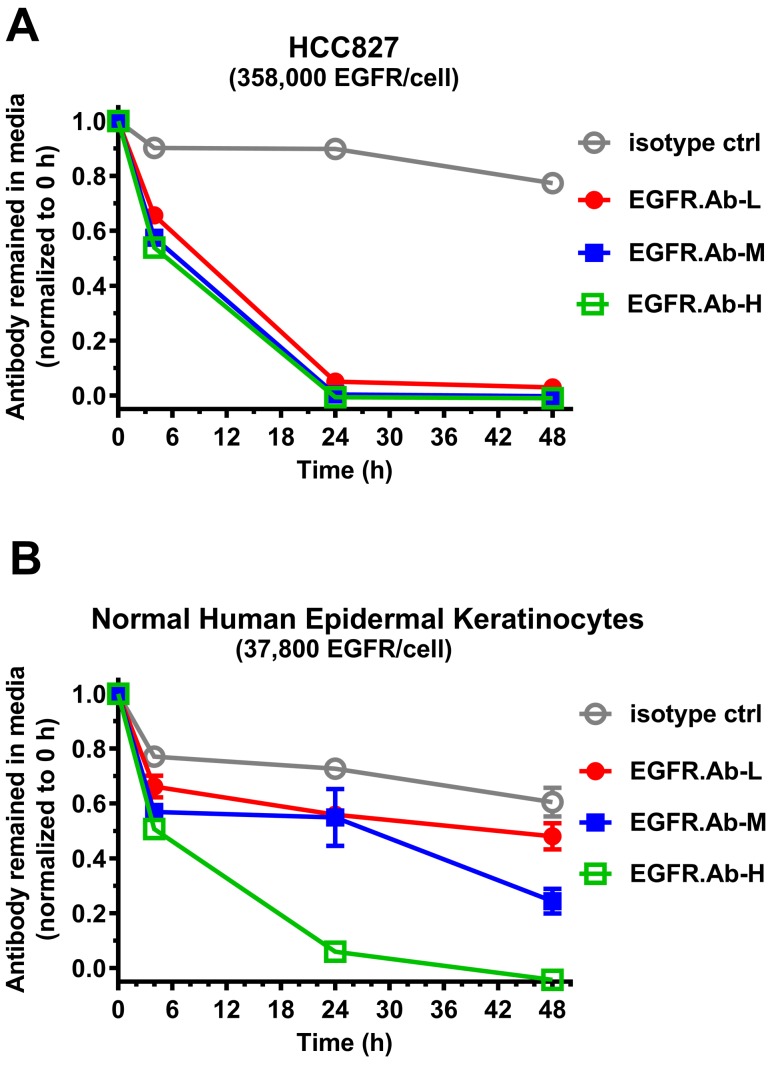
Low affinity EGFR antibody demonstrated differential uptake by cancer cells and normal human epidermal keratinocytes NSCLC cell line HCC827 (**A)** or normal human epidermal keratinocytes (**B)** were incubated with the indicated EGFR affinity variant antibodies or isotype control antibody. The amount of antibody remaining in the media was determined by ELISA at the indicated time points and then normalized to the value at time zero. EGFR.Ab-L (low affinity), EGFR.Ab-M (medium affinity), EGFR.Ab-H (high affinity). Each treatment was done in triplicate.

Next, we developed an *in vitro* antibody binding/uptake assay to enable selection of an antibody with optimized affinity such that binding and uptake by EGFR occurs more efficiently on overexpressing cancer cells compared to normal cells. Cutaneous toxicity is frequently associated with EGFR-targeting therapies and hence we used normal human epidermal keratinocytes as an *in vitro* model system to access EGFR-mediated toxicity. For EGFR overexpressing cells, we used non-small cell lung cancer cell line HCC827 which has ∼358,000 receptors per cell (Table 2). Cells were incubated with EGFR antibodies or isotype control antibodies. At various time points, the concentration of free unbound antibodies remaining in the culture media was determined as described in Materials and Methods. Of the three EGFR antibodies incubated with HCC827, there was no difference in their binding or uptake kinetics by the HCC827 cells (Figure 2A). All three EGFR antibodies were nearly 100% taken up by HCC827 cells after 24 h incubation regardless of their affinities. With the normal human keratinocytes, differential antibody uptake kinetics was observed (Figure 2B). The high affinity antibody EGFR.Ab-H was efficiently taken up by the keratinocytes after 24 h of incubation. In contrast, a substantial percentage of the medium and low affinity EGFR antibodies, EGFR.Ab-M and EGFR.Ab-L, respectively, still remained in the culture media even after 48 h. At the end of the assay, EGFR.Ab-L has the highest amount (∼50%) of free antibody remaining in the media at level similar to that of the isotype control (Figure 2B). Therefore, EGFR.Ab-L was chosen as the antibody backbone for site-specific conjugation to generate the low affinity EGFR ADC, RN765C.

**Table 2:** EC50 values on cancer cell lines and normal human keratinocytes

		EC50 (nM)					
Cell line (tumor type)	EGFR (receptor/cell)	RN765C	Free payload	Neg ctrl ADC	Cetuximab	EGFR. Ab-H	EGFR. Ab-L
MDA-MB-468 (Breast)	++++	0.013	0.020	25.82	>233 (PR)	>233 (PR)	>233 (PR)
HCC827 (NSCLC)	+++ (358,000)	0.030	0.293	166.04	>233 (PR)	>233 (PR)	>233 (PR)
A431 (Epidermoid)	+++ (183,900)	0.029	0.036	>200 (PR)	>200 (PR)	N.D.	N.D.
FaDu (HNSCC)	++ (53,800)	0.806	0.060	34.33	>233 (PR)	>233 (PR)	>233 (PR)
BxPC3 (Pancreas)	++ (51,400)	2.892	0.143	59.59	>233 (NR)	>233 (NR)	>233 (NR)
NCI-H1975 (NSCLC)	+/++ (48,800)	5.364	0.067	138.56	>200 (NR)	N.D.	N.D.
HT29 (CRC)	+/++ (36,100)	71.937	0.032	107.16	>226 (NR)	N.D.	N.D.
NCI-H1650 (NSCLC)	+/++ (32,500)	107.775	0.617	>167 (NR)	>167 (NR)	N.D.	N.D.
SW620 (CRC)	-(0)	>200 (PR)	0.085	>200 (PR)	>200 (NR)	N.D.	>200 (NR)
Human Epidermal Keratinocyte (Normal)	+/++ (37,800)	72.618	0.098	130.97	121.29	N.D.	>266 (PR)

Cytotoxicity assays of RN765C, EGFR antibodies, free payload and negative control ADC (Neg ctrl ADC) were performed as described in Materials and Methods. EC50 (defined as 50% growth inhibition) is the concentration of the cytotoxic agent that will yield 50% relative viable cell number as compared to the unconjugated isotype control antibody-treated wells. Each treatment was done in triplicate. NR = no response. PR = partial response, the treatment induced some cytotoxicity but it did not reach 50% growth inhibition level even at the highest concentration tested. Symbol “>” indicates 50% growth inhibition was not reached even at the highest concentration used as indicated. N.D.= not determined.

### RN765C exerts potent *in vitro* killing activity on tumor cells but not normal human keratinocytes

**Figure 3:**
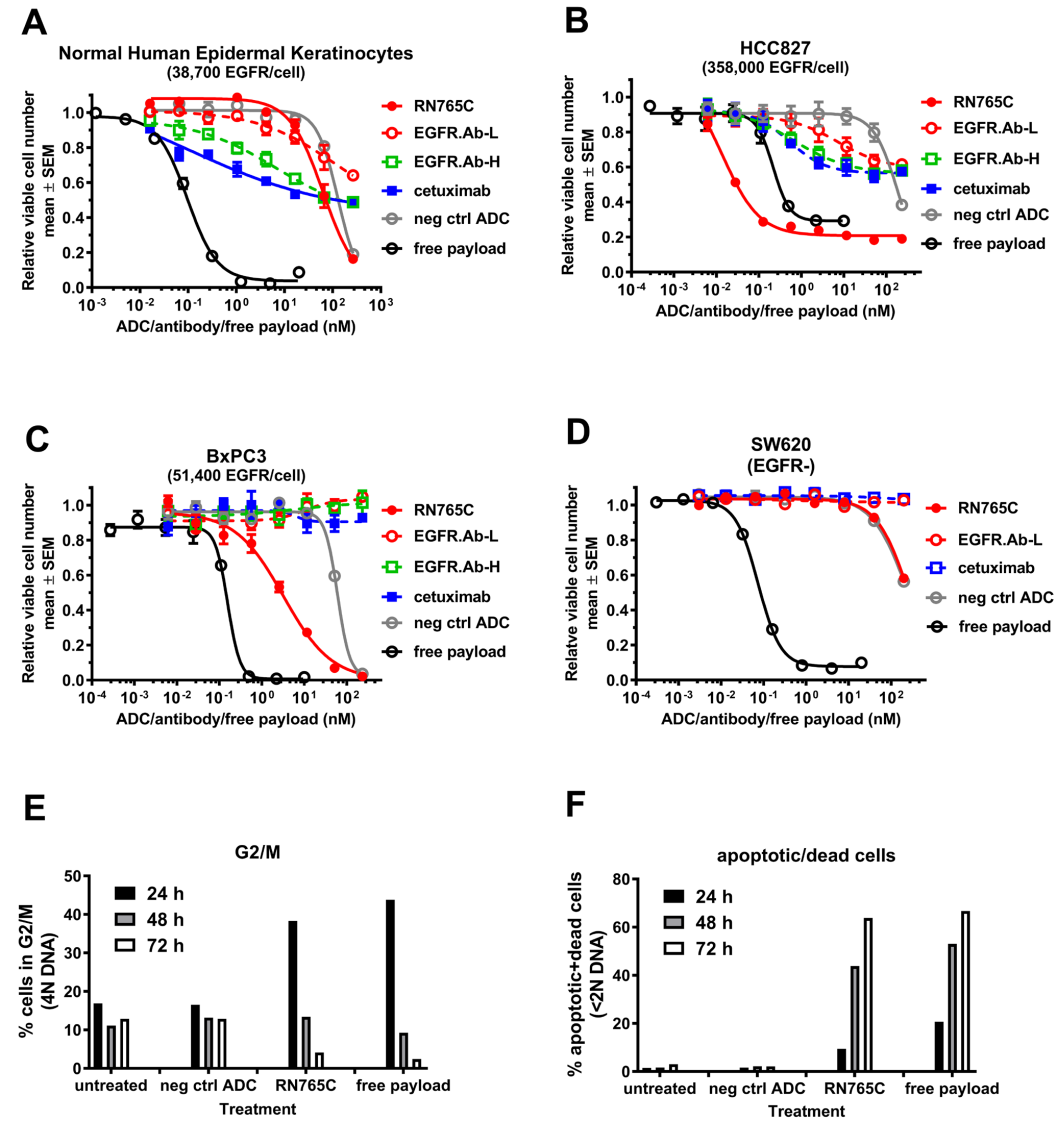
RN765C, a low affinity EGFR ADC, has potent cytotoxicity towards cancers cells and it induces mitotic arrest and cell death Normal human epidermal keratinocytes (**A)**, NSCLC cell line HCC827 (**B)**, pancreatic cancer cell line BxPC3 (**C)** and EGFR negative colorectal cancer cell line SW620 (**D),** were exposed to the indicated agents over a range of concentrations for 4-5 days. Relative viable cell number was determined at the end of treatment. Free payload and negative control ADC (neg ctrl ADC) generated with the same conjugation site and linker payload were included as references. Unconjugated EGFR antibodies: cetuximab, EGFR.Ab-L (low affinity) and EGFR.Ab-H (high affinity). (**E)** and (**F)**, MDA.MB.468 cells were exposed to 2 µg/mL RN765C, 2 µg/mL neg ctrl ADC or 5 nM free-payload PF-06380101 for the indicated duration. At the end of treatment, cells were harvested and DNA content was measured to determine the % of G2/M phase cells (**E**) and % of apoptotic and dead cells (**F**).

RN765C is generated by conjugating AcLys-VC-PABC-PF-06380101 [10, 11] to the transglutaminase tag (GGLLQGPP) located at the C-terminus of the antibody light chain (see Materials and Methods), providing a maximum DAR (Drug-Antibody-Ratio) of 2. The transglutaminase tag allows precise enzymatic conjugation using bacterial transglutaminase to generate a covalent linkage between the glutamine in the tag GGLLQGPP and the AcLys linker as previously reported [9]. The AcLys cleavable linker was chosen to improve the stability of the conjugate in circulation [11]. The payload PF-06380101 [10] is a potent anti-mitotic agent that inhibits tubulin polymerization. The chemical structure of RN765C is shown in Supplementary Figure 1. We compared the sensitivity of normal human epidermal keratinocytes to RN765C, the low affinity antibody EGFR.Ab-L, the high affinity antibodies EGFR.Ab-H and cetuximab. Over the range of antibody concentrations tested, the low affinity antibody, EGFR.Ab-L, is less cytotoxic to keratinocytes than cetuximab and EGFR.Ab-H (Figure 3A). Even with the ADC, RN765C, it did not show higher cytotoxicity than both cetuximab and EGFR.Ab-H at concentrations up to 67 nM. Non-target mediated ADC uptake by pinocytosis may have partly contributed to the RN765C cytotoxicity at concentration ≥ 67 nM since a negative control conjugate consisting of a non-binding antibody conjugated in the same manner as RN765C also induced significant keratinocyte killing at high concentration (Figure 3A). The low toxicity of RN765C is not due to intrinsic insensitivity of keratinocytes to the payload PF-06380101 as free payload has an EC50 of 0.098 nM for keratinocytes (Figure 3A and Table 2). RN765C, on the other hand, killed EGFR overexpressing HCC827 cells readily with an EC50 of 0.030 nM, even more potent than free payload (Figure 3B and Table 2). In contrast, both cetuximab and EGFR.Ab-H were unable to kill more than 50% of the HCC827 cells even at concentration > 200 nM. Notably the low affinity EGFR.Ab-L still retains EGFR signal blocking ability, as demonstrated by the 30-40% growth inhibition of HCC827 cells at antibody concentration ≥ 10 nM (Figure 3B). BxPC3, a pancreatic cell line that has moderate EGFR expression (51,400 receptors/cell), is completely non-responsive to naked EGFR antibodies but still sensitive to RN765C with an EC50 of 2.89 nM (Figure 3C and Table 2). For EGFR negative colorectal cancer cell line SW620, RN765C had little effect and behaved just like the negative control ADC (Figure 3D and Table 2) confirming the specificity of RN765C. A number of cancer cell lines that are insensitive to cetuximab can be killed by RN765C readily with EC50 in the single digit nanomolar to sub-nanomolar range provided that sufficient receptors are present (Table 2). These data demonstrated that RN765C is quite potent against tumor cells with medium to high EGFR expression (typically > 50,000 receptors/cell) but is less cytotoxic to cells with low EGFR expression, such as normal human keratinocytes, HT29 and NCI-H1650.

### RN765C induces mitotic arrest in EGFR-expressing cells

The presumed mechanism through which RN765C elicits cytotoxicity is by intracellular release of the PF-06380101 payload, which results in the disruption of microtubule polymerization leading to mitotic arrest, apoptosis and cell death. To confirm this mechanism, EGFR-positive MDA.MB.468 cells were incubated with RN765C, negative control ADC and free payload PF-06380101 for the indicated time (Figure 3E and 3F). Cell cycle phase was determined by DNA content as described in Materials and Methods. At 24 h post treatment, significant accumulation of cells in the G2/M phase (4N DNA content), an indication of mitotic arrest, was observed in the RN765C and free payload treated samples. As treatment time increased, a reduction in the % of G2/M phase cells was observed. This was then accompanied by an increase in % of apoptotic and dead cells (<2N DNA content) which peaked at 72 h. This suggested that the G2/M arrested cells were converted to apoptotic and dead cells over time, consistent with the mechanism of the payload. In contrast, there was very little change in the % of G2/M and apoptosis/dead cells in the untreated and negative control ADC samples over time. Since antibody dependent cell cytotoxicity (ADCC) is an important mechanism for approved EGFR monoclonal antibodies, next we tested if RN765C can mediate ADCC. We found that RN765C retained robust ADCC with potency similar to cetuximab (Supplementary Figure 2). Altogether, it demonstrated that RN765C possesses multiple mechanisms to mediate tumor cell killing.

### RN765C is highly efficacious in multiple solid tumor xenograft models

**Figure 4:**
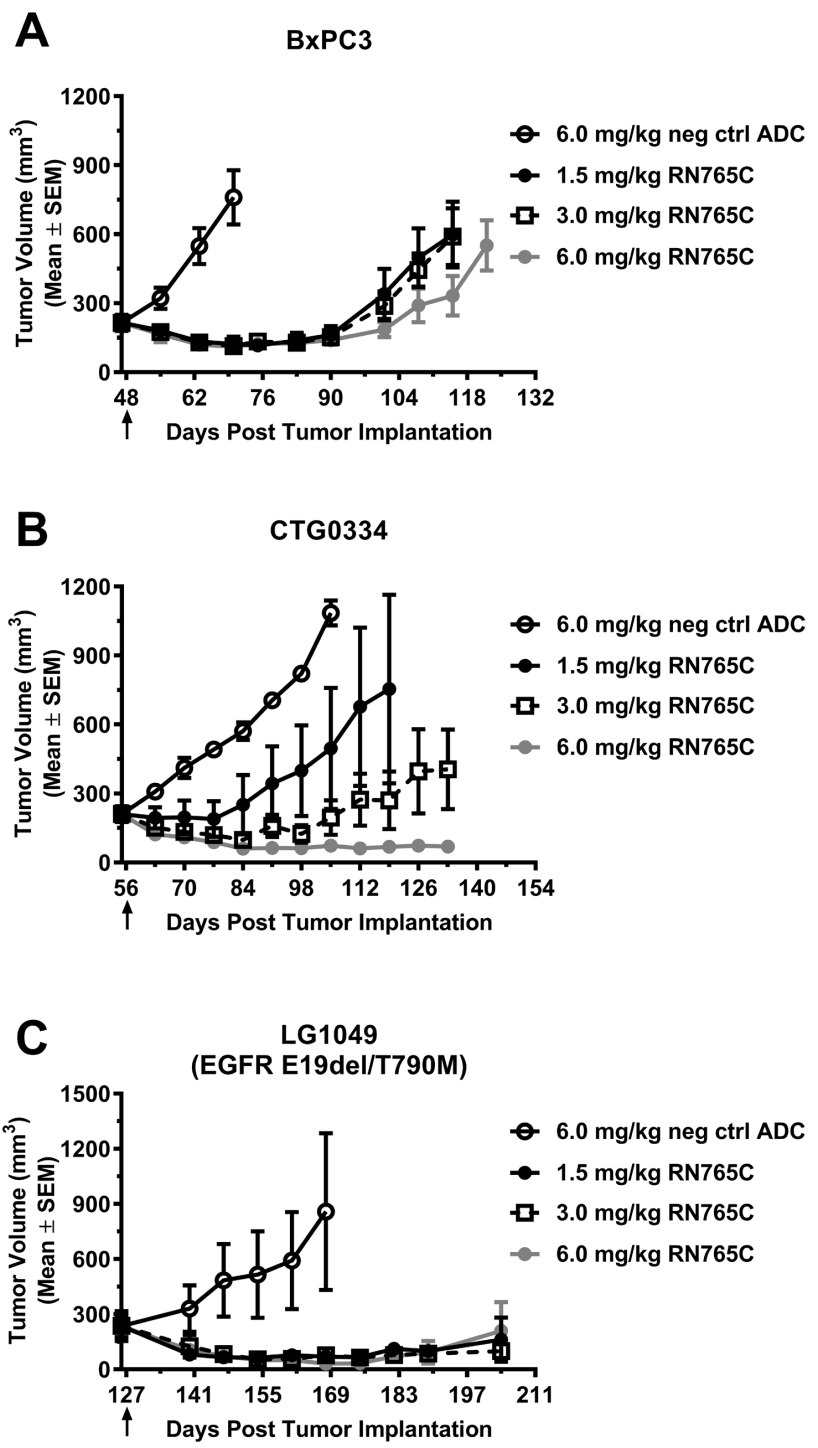
A single dose of RN765C is efficacious in multiple xenograft models (**A)** Pancreatic cell line model BxPC3. (**B)** Colorectal cancer PDX model CTG0334. (**C)** NSCLC PDX model LG1049 which harbors EGFR mutation E19del and T790M. Black arrows indicate the time at which treatment was given. 5 mice per group.

The *in vivo* efficacy of RN765C was tested on multiple tumor types that were originated from pancreatic cancer cell line BxPC3 or PDX models of colorectal cancer (CTG0334) and lung cancer (LG1049). One dose of 1.5 mg/kg RN765C treatment resulted in sustained tumor growth inhibition/regression in these models (Figure 4). The negative control ADC failed to control tumor growth on these tumor models at 6 mg/kg, the highest dose tested in these studies. Notably, RN765C is very potent towards the non-small cell lung cancer PDX LG1049 which carries the EGFR exon 19 deletion and T790M mutation, the latter is known to drive resistance to first generation TKIs (e.g. erlotinib and gefitinib) of EGFR. One dose of 1.5 mg/kg RN765C was able to induce tumor regression for over 60 days (Figure 4C). This data illustrated that RN765C is efficacious in multiple solid tumor types.

### RN765C is more efficacious than standard of care in multiple non-small cell lung cancer PDX models

**Figure 5:**
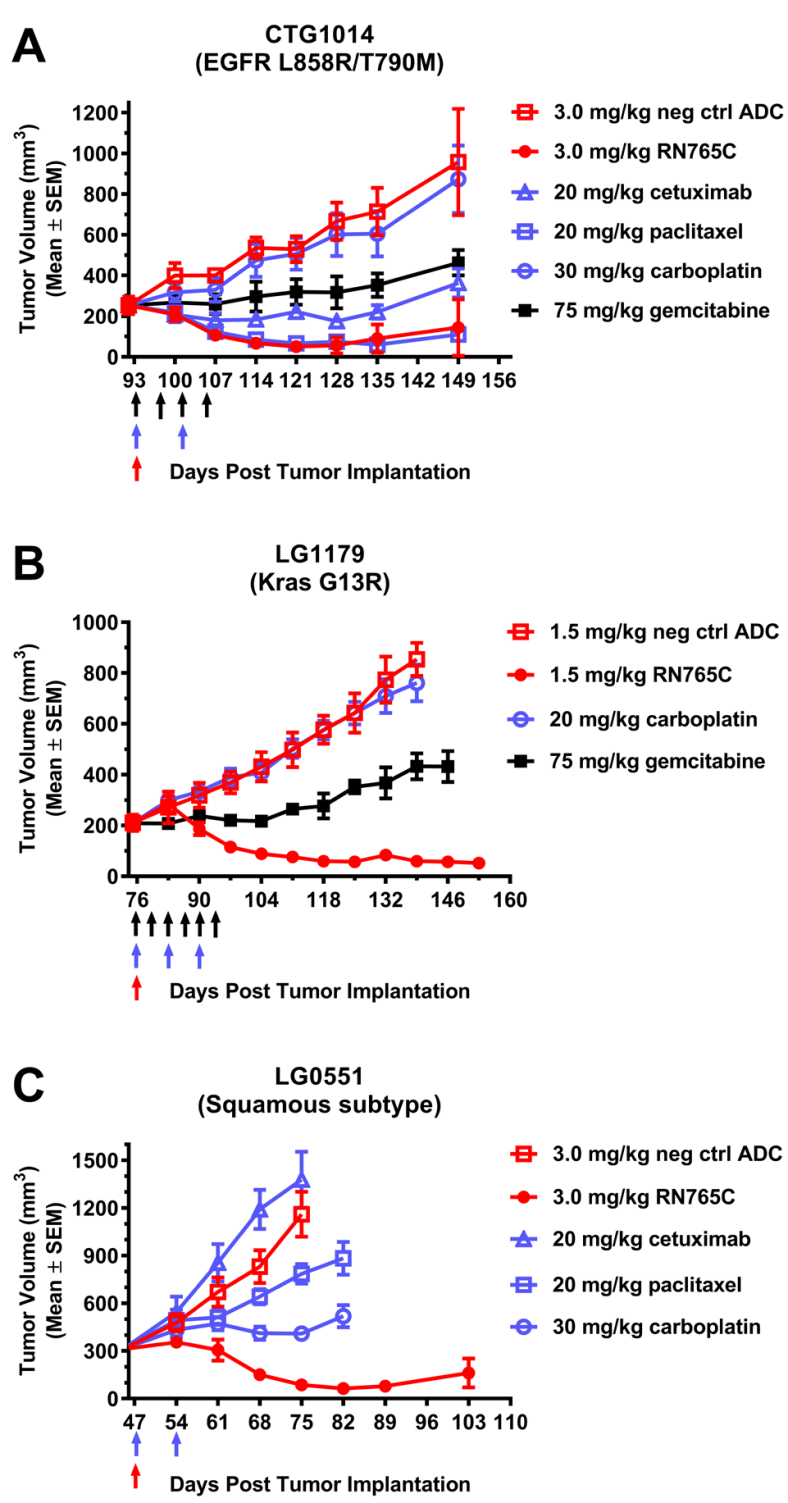
RN765C is more efficacious than standard of care in multiple NSCLC PDX models (**A)** CTG1014 PDX model carries EGFR L858R/T790M mutations, 4 mice per group. (**B)** LG1179 is a Kras G13R mutant PDX model, 4 mice per group. (**C)** LG0551 is a squamous cell carcinoma PDX model, 5 mice per group. Colored arrows represent the dosing frequency of the treatment with the corresponding symbol colors. In brief, cetuximab and paclitaxel were given once a week for 2 weeks; carboplatin was given once a week for 2 weeks in (**A**) and (**C**), for 3 weeks in (**B**); gemcitabine was given twice a week for 2 weeks in (**A**), for 3 weeks in (**B**).

Non-small cell lung cancer is an important tumor type for EGFR targeted therapy and many NSCLC express high level of EGFR. Next, we compared the anti-tumor efficacy of RN765C to standard of care therapies in three NSCLC PDX models. In CTG1014, which carries EGFR L858R/T790M mutations, a single dose of 3.0 mg/ml RN765C was sufficient to induce tumor regression with similar efficacy as two doses of paclitaxel and was more efficacious than multiple doses of gemcitabine, carboplatin or cetuximab (Figure 5A). In LG1179 (Kras G13R; Kras mutants are typically non-responsive to EGFR TKIs), one 1.5 mg/kg dose of RN765C resulted in sustained tumor regression while multiple doses of carboplatin were ineffective and gemcitabine only moderately slowed down tumor growth (Figure 5B). For the squamous PDX model LG0551, a single dose of 3.0 mg/kg RN765C once again outperformed two doses of carboplatin, paclitaxel and cetuximab (Figure 5C). Taken together, it demonstrated that RN765C is more efficacious than standard of care in EGFR positive NSCLC models regardless of their EGFR, Kras mutation status and histological subtype.

## DISCUSSION

We described here a novel low affinity anti-EGFR ADC, RN765C, designed to have potent anti-tumor activity in tumors with medium to high EGFR expression. RN765C is able to utilize three mechanisms to inhibit tumor growth: 1) active cell killing mediated by its potent payload PF-06380101, 2) blocking of EGFR signaling pathway due to ligand blocking property of its parental antibody, and 3) antibody dependent cell cytotoxicity provided by human IgG1 backbone (Table 2, Figure 3 and Supplementary Figure 2). EGFR has high prevalence in multiple solid tumor types, such as head and neck cancers, NSCLC and colorectal cancers, and is an attractive target for cancer therapy. However, it is also widely expressed on a number of normal epithelial tissues such as skin and gastrointestinal tissue and thus represents a toxicity risk. Therefore, the design of an anti-EGFR ADC required a careful balance between efficacy and safety. Over the past decade, multiple strategies have been developed to target EGFR expressing tumors more effectively while keeping on target normal tissue toxicity to a minimum. One approach is to develop pro-antibody drug conjugates (PDC or probody) in which the antigen binding sites of the antibody moiety are masked by a peptide until locally activated by proteases commonly found in tumor microenvironment [12, 13]. Another strategy is to design an EGFR ADC targeting a conformation epitope that is more frequently presented in EGFR amplified or overexpressed tumors than normal tissues [14-16]. Indeed, early clinical data of this approach indicated the absence of conventional EGFR inhibitors mediated toxicities [17-19]. Others chose to target the unique epitope found in the tumor specific extracellular domain truncation mutant EGFRvIII, which is commonly present in glioblastoma, to totally avoid the wild-type receptors [20-22]. Some groups use a mixture of two or more EGFR antibodies with non-overlapping epitope to drive receptor degradation and hence halt EGFR signaling [23-25]. We took multiple approaches to mitigate the safety concern while maintaining tumor targeting efficacy of RN765C. Most approved ADCs or ADCs under clinical development have been manufactured through cysteine disulfide bond or lysine based conjugations, producing heterogeneous mixtures of ADC species (DAR range of 0-8) with an average DAR around 3-4 [26]. A broad distribution of DAR can negatively impact both efficacy and safety profiles: low DAR species reduce the potency of ADC while high DAR species are prone to aggregation, increased clearance rate and premature release of the toxic payload during circulation leading to increase off-target toxicity [27]. To circumvent this problem, we and others have developed various site-specific conjugation technologies to generate a more homogeneous ADC with increased stability in the circulation [9, 27-29]. RN765C uses transglutaminase-mediated site-specific conjugation to enable production of a nearly homogeneous EGFR ADCs carrying two payloads (DAR 2) [9, 30]. By making a homogeneous ADC with a DAR of 2, eliminating high DAR species, and utilizing stable isopeptide linkage and incorporating an AcLys-VC-PABC linker that was shown to be more stable in circulation [11, 30, 31], we aimed to reduce both off-target toxicity due to unintended payload release in circulation as well as on-target toxicity due to delivery of high DAR species to EGFR expressing normal tissue. Furthermore, we selected a low affinity EGFR antibody as the backbone for RN765C to achieve differential binding on normal cells *versus* tumor cells expressing medium to high levels of EGFR. The low affinity nature of RN765C (*K*_D_ = 338 nM at 37°C) reduces its toxicity on low EGFR expressing cells such as normal human keratinocytes, which have ∼ 38,000 EGFR/cell (Table 2). For human keratinocytes it requires ∼ 70 nM of RN765C to achieve 50% of cell killing *in vitro* (which is partly driven by non-receptor mediated pinocytosis) whereas the same level of killing can be achieved with sub-nanomolar to low nanomolar of RN765C in many tumor cell lines expressing > 50,000 EGFR/cell (Figure 3A, 3B and 3C, and Table 2). Aside from the potential to differentially target tumor cells, the low affinity and fast dissociation rate constant (*k*_*d*_ = 0.21 s^-1^) nature of RN765C may also facilitate tumor penetration as compared with high affinity ADCs [32, 33].

RN765C is highly efficacious *in vivo*, with tumor regression generally achieved with single injection of RN765C at a dose of 1.5 - 3.0 mg/kg (Figures 4 and 5). Efficacy is seen in pancreatic, colorectal, and lung cancer models regardless of their Kras mutation and TKI resistant EGFR mutation status. We also found that RN765C is generally more efficacious than standard of care in PDX models of NSCLC, an important indication for EGFR-targeted therapies. For instance, in the NSCLC cancer PDX models LG1179 (Kras G13R) and LG0551 (squamous cell carcinoma), a single RN765C treatment at 1.5 mg/kg or 3.0 mg/kg, respectively, induces sustained tumor regression, while multiple doses of gemcitabine, carboplatin and paclitaxel either have no effect (carboplatin in LG1179) or only leads to tumor growth inhibition (Figure 5B and 5C). This is encouraging because Kras mutated NSCLC and colorectal cancers are non-responsive to EGFR TKIs or monoclonal antibody therapies. In addition, RN765C is able to induce sustained tumor regression in the LG0551 model which is insensitive to cetuximab treatment.

To date, a total of four ADCs have been approved by the Food and Drug Administration for treatment of cancers. Among them three (gemtuzumab ozogamicin, brentuximab vedotin and inotuzumab ozogamicin) are for hematologic malignances and only one (ado-trastuzumab emtansine) is for solid tumor indication [6]. As most solid tumor antigens have normal tissue expression, one of the major obstacles for ADCs in solid tumors is to deliver sufficient toxic payload to tumor cells without causing serious normal tissue toxicity. In this report, we explored the approach of using a low affinity EGFR ADC to preferentially target tumor cells that express medium to high level of EGFR. Despite the low affinity, RN765C is quite potent and has better anti-tumor activity compared to standard of care in multiple solid tumor models, including those that are non-responsive to naked EGFR antibody therapies or TKIs. Our data suggest that RN765C may have the potential to target a broader population than the current approved EGFR-targeted therapies and thus warrants further investigation.

## MATERIALS AND METHODS

### Cell lines, antibodies and reagents

Human cancer cell lines A431, BxPC3, FaDu, HCC827, HT-29, NCI-H1650, NCI-H1975 and MDA-MB-468 were directly obtained from and authenticated by American Type Culture Collection and cultured in DMEM+10% FBS (fetal bovine serum). To ensure authentication and consistency throughout the study, only low-passage cells (< 2 months old post thawing) were used in the experiments. Normal human epidermal keratinocytes (NHEK) were purchased from Lonza and cultured in KBM-Gold complete medium (Lonza). Cetuximab biosimilar was made by cloning the DNA encoding this antibody into in-house expression plasmid, transiently expressed in HEK293 cells and then purified with Protein-A MabSelect SuRe columns (GE Life Sciences). Monomeric 8xHis-tagged human EGFR extracellular domain (ECD) protein was generated from cloning amino acid residues #25-642 of the human EGFR to an expression vector with an 8xHis-tag at the C-terminus. The 8xHis-tagged protein was expressed in HEK293 cells and purified with standard techniques using nickel-NTA column. Monomeric 8xHis-tagged cynomolgus EGFR ECD comprised of amino acid residues #25-645 was generated similarly. Gemcitabine (GEMZAR) was purchased from Eli Lily and company. Paclitaxel and carboplatin were purchased from Sigma-Aldrich.

### Generation of EGFR antibodies

Anti-EGFR monoclonal antibody mAb-D was derived from immunization of Balb/c mice using A431 cells and hybridomas were generated using standard techniques. The mouse variable domains of mAb-D were cloned into human IgG1/κ constant domains to generate a chimeric antibody mAb-D.hIgG1. To generate affinity variants of anti-EGFR antibodies, mAb-D was humanized by CDR grafting and cloned into human IgG1/κ constant domains to create the parental antibody EGFR.Ab-M. High affinity variant, EGFR.Ab-H, and low affinity variant, EGFR.Ab-L, of the parental antibody were generated by screening a library containing single amino acid mutations of the light-chain variable domain.

### Site-specific ADC conjugation to generate RN765C

To facilitate site-specific conjugation of cytotoxic agent, a transglutaminase tag consisting of amino acid sequence GGLLQGPP was added to the C-terminus of the light-chain constant domain of the low affinity antibody EGFR.Ab-L and the heavy chain hinge residue lysine 222 was mutated to arginine (K222R). The resulting EGFR antibody, named RN765, was transiently expressed in HEK293 cells, and then purified with Protein-A MabSelect SuRe columns (GE Life Sciences).

ADC, RN765C, was generated from linker-payload AcLys-VC-PABC-PF-06380101 [10, 11] and naked antibody RN765. The antibody concentration was adjusted to 5 mg/mL in buffer containing 25 mM Tris-HCl, pH 8.0, 150 mM sodium chloride. Linker-payload [11] was added in a 10-fold molar excess over antibody, the conjugation reaction was initiated by addition of 2% (w/v) bacterial transglutaminase (Ajinomoto Activa TI) and incubated with gentle shaking at 37°C for 24 hours. RN765C was purified from the reaction mixture as described previously [9]. The final product DAR (Drug-Antibody-Ratio) was typically 1.93-2.0.

### EGFR antibody affinity determination

The binding affinities of recombinant anti-EGFR antibodies were measured on a surface plasmon resonance (SPR) Biacore™ T200 biosensor (GE Life Sciences). An anti-human IgG Fc sensor surface was prepared at 25°C with a running buffer of 10 mM HEPES, 150 mM NaCl, 0.05% Tween-20, pH 7.4. All surfaces of a Biacore CM4 sensor chip were activated with a 1:1 (v/v) mixture of 400 mM 1-ethyl-3-(3-dimethylaminopropyl)carbodiimide hydrochloride and 100 mM N-Hydroxysuccinimide for 7 minutes at a flow rate of 10 µL/min. Anti-human IgG Fc (SouthernBiotech) was diluted to 50 µg/mL in 10 mM acetate pH 4.5 and injected in all flow cells for 7 minutes at 20 µL/min. All flow cells were blocked with 100 mM ethylenediamine in 200 mM borate buffer pH 8.

All interaction analysis was performed at 37°C using a running buffer of 10 mM HEPES, 150 mM NaCl, 0.05% Tween-20, 1 mg/mL BSA, pH 7.4. All reagents were diluted into running buffer prior to analysis. Antibodies (IgG) were captured at 10 µg/mL onto flow cells 2, 3 and 4 at a flow rate of 10 µL/min for 2 minutes. Different antibodies were captured in each flow cell. Flow cell 1 was used as a reference surface. Following capture of antibodies, analyte (buffer or 8xHis-tagged monomeric human or cynomolgus EGFR extracellular domain) was injected at 30 µL/min for 2 minutes. After the analyte injection, dissociation was monitored for 10 minutes followed by regeneration of all flow cells with three 1 minute injections of 75 mM Phosphoric Acid. For each captured antibody the following analyte injections were performed: buffer, 0.08 nM, 4 nM, 20 nM, 100 nM and 500 nM EGFR. The data were fit to a *1:1 Langmuir with mass transport* model to determine the association rate constants (*k*_a_) and dissociation rate constants (*k*_d_) values using Biacore T200 Evaluation Software 2.0. The equilibrium dissociation constant (*K*_D_) and t_1/2_ (half-life) values for each antigen/antibody interaction were derived from *K*_D_ = *k*_d_/*k*_a_ and t_1/2_ = ln 2/*k*_d_, respectively.

### Antibody uptake assay

Non-small cell lung cancer cells HCC827 and normal human epidermal keratinocytes were seeded at ∼300,000 cells/well onto 12-well plate in growth medium. Cells were allowed to grow at 37°C in a humidified CO_2_ incubator. After a few days, when cells were near confluent, old media were carefully removed by aspiration. Two mL of DMEM + 2%FBS (for HCC827) or 2 mL of KBM-Gold medium (for keratinocytes) containing 0.05 µg/mL of EGFR antibodies or isotype control antibody were added to wells. A 300 µL of media was then withdrawn from each well at 1 min, 4 h, 24 h and 48 h post addition of the antibodies and the concentrations of antibodies remaining in the media were measured by ELISA.

### EGFR density determination

Receptor density on cell lines was determined using Quantum Simply Cellular (QSC) anti-human IgG kit (Bangs Laboratories) according to manufacturer’s protocol. Briefly, cells were harvested from culture flask using CellStripper non-enzymatic dissociation reagent (Fisher Scientific). After washing with FACS buffer (PBS+3% BSA), cells were suspended with FACS buffer at 1x10^6^ cells/mL. One hundred µL of cells was added to a microcentrifuge tube. Cells were then incubated with 3 µg of high affinity EGFR antibody (cetuximab or EGFR.Ab-H) for 45 min at 4°C on a tube rotator. After washing three times with 1 mL FACS buffer, 100 µL of 1:25 dilution of PerCP conjugated goat anti-mouse F(ab’)2 specific (use with cetuximab; Jackson Immuno Research) or goat anti-human F(ab’)2 specific (use with EGFR.Ab-H; Jacson Immuno Research) secondary antibody was added to cell pellet and further incubated at 4°C for 45 min on the tube rotator. Cells were washed three times and acquired on a flow cytometer immediately. The five standard bead populations from the QSC kit were prepared and acquired in the same way as the cell samples. Mean fluorescence intensity (MFI) values for the 5 bead populations and cells were recorded in the manufacturer provided lot-specific QuickCal template. The MFI values from the bead populations, with known ABC (Antibody Binding Capacity), were used to generate a standard curve to assign ABC for cells samples.

### Cytotoxicity assay

Human tumor cells or normal primary cells were seeded onto white-walled 96-well plate at 1500 cells/well in DMEM + 10% FBS (unless specified) or at 1300 cells/well for normal human epidermal keratinocytes in KBM-Gold complete medium. Plates were then incubated at 37°C in a humidified CO_2_ incubator overnight. Next day, cells were treated with serially diluted RN765C, negative control ADC, unconjugated anti-EGFR antibodies or free payload PF-06380101 at the desired concentrations in their growth media. Cells were further incubated at 37°C for 5 days, or 4 days for HCC827. After treatment, medium in each well was discarded and CellTiter-Glo^®^ (Promega, Madison WI) viability assays were performed. Luminescence signals were detected on the SpectraMax M5 plate reader (Molecular Devices). Relative viable cell number was determined by normalizing readings from each well to that of unconjugated isotype control antibody-treated wells. Each treatment was done in triplicate. Dose-response curves were generated and fitted with non-linear regression to a four-parameter dose-response equation using GraphPad Prism 6.03. EC50 values (defined as the concentration of antibody or ADC that resulted in 50% growth compared to unconjugated isotype control treated samples, i.e. relative viable cell number (y-axis) = 0.5) were then generated from the fitted curves. Test agent that did not induce ≥ 50% killing were further classified into NR (No response) or PR (partial response, the agent induces some killing but it did not reach 50%).

### Cell cycle analysis

MDA-MB-468 cells were seeded in 6-well plates at 0.2 to 1 million cells per well in growth medium (DMEM+10%FBS). The following day, medium was removed and replaced with 2 mL fresh growth media containing the following ADCs or a proprietary microtubule inhibitor (PF-06380101): 2 µg/mL RN765C, 2 µg/mL negative control ADC (a non-binding isotype control ADC) or 5 nM PF-06380101. Cells were incubated at 37°C in a humidified CO_2_ incubator for 24, 48 or 72 hours. A no treatment control was included in each time point. At the end of treatment, both the medium and cells were harvested and combined into a collection tube. Samples were then processed with CycleTEST Plus DNA Reagent Kit (BD Biosciences) according to manufacturer’s protocol. DNA content data were acquired within 1 hour on a LSRII Cell Analyzer (BD Biosciences). 30,000 events were collected for each sample and cell cycle phase analysis was performed on FlowJo v.10 (FlowJo, LLC).

### *In vivo* studies

All animal studies were conducted in an AAALAC accredited facility. All procedures performed on these animals were in accordance with regulations and established guidelines and were reviewed and approved by Pfizer Institutional Animal Care and Use Committee. Four or five animals per cohort were used in all the efficacy studies. For BxPC3 xenograft model, 2 million cells were implanted subcutaneously into 5-6 weeks old female CB17/SCID mice (The Jackson Laboratory) until the tumor sizes reached 250-300 mm3 before treatment started. Non-small cell lung carcinoma PDX models LG1049 (which harbors the EGFR Exon19del/T790M mutations), LG1179 (Kras G13R mutation, paclitaxel resistant) and LG0551 (squamous carcinoma) were acquired from The Jackson Laboratory. The NSCLC PDX model CTG1014 (EGFR L858R/T790M mutations) and colorectal cancer PDX model CTG0334 (Kras wild-type) were acquired from Champion Oncology. For PDX models, 1-2 mm3 of tumor fragments were implanted subcutaneously into the lateral flanks of female CB17/SCID mice from Taconic. Animals were randomized by tumor sizes once they reached ∼300 mm3 or otherwise indicated, and RN765C and other agents were administered through bolus tail vein injection. Tumor volume was calculated with the following formula: Tumor volume = (length x width ^2^) / 2. Animals were humanely euthanized before their tumor volumes reached 2000 mm^3^.

## SUPPLEMENTARY MATERIALS FIGURES



## Abbreviations

ADC: antibody-drug conjugate
TKI: tyrosine kinase inhibitors
PDC: pro-antibody drug conjugates
DAR: drug-antibody ratio
ECD: extracellular domain
ADCC: antibody dependent cell cytotoxicity
PDX: patient-derived xenografts
NSCLC: non-small cell lung cancer
