# Supplementary material for: RN765C, a low affinity EGFR antibody drug conjugate with potent anti-tumor activity in preclinical solid tumor models
Source: Oncotarget. 2018 Sep 11;9(71):33446–58. doi: 10.18632/oncotarget.26002 (PMC6173368; doi:10.18632/oncotarget.26002)
Supplement: Supplementary file 1 [file oncotarget-09-33446-s001.pdf]

RN765C, a low affinity EGFR antibody drug conjugate with potent anti-tumor activity in preclinical solid tumor models

SUPPLEMENTARY MATERIALS

Antibody dependent cell cytotoxicity (ADCC) assay

Target expressing FaDu cells were seeded at 10,000 cells/well in 100 µL RPMI+5% FBS onto 96-well plate and incubated at 37°C. Purified CD56+ NK cells (HemaCare) were thawed and incubated at 37oC overnight in X-Vivo15 medium (Lonza) supplemented with 5% human AB serum. On the following day, a mixture containing 100,000 NK cells (E:T=10:1) plus 20 µg/mL antibodies or ADC in 100 µL RPMI+5% FBS were added to the wells. The plate was incubated at 37°C for 4 hours. All treatments were done in duplicate and wells with target cells alone or target cells plus NK cells were included as controls. ADCC induced target cell death was measured by LDH (Lactate Dehydrogenase) release using CytoTox 96 non-radioactive cytotoxicity assay kit (Promega) according to manufacturer's protocol. Percentages (%) of specific lysis were calculated with the following formula:

% specific lysis = (treatment induced LDH release - target cell spontaneous LDH release - effector cell spontaneous LDH release) / (target cell maximum LDH release - target cell spontaneous LDH release) x 100

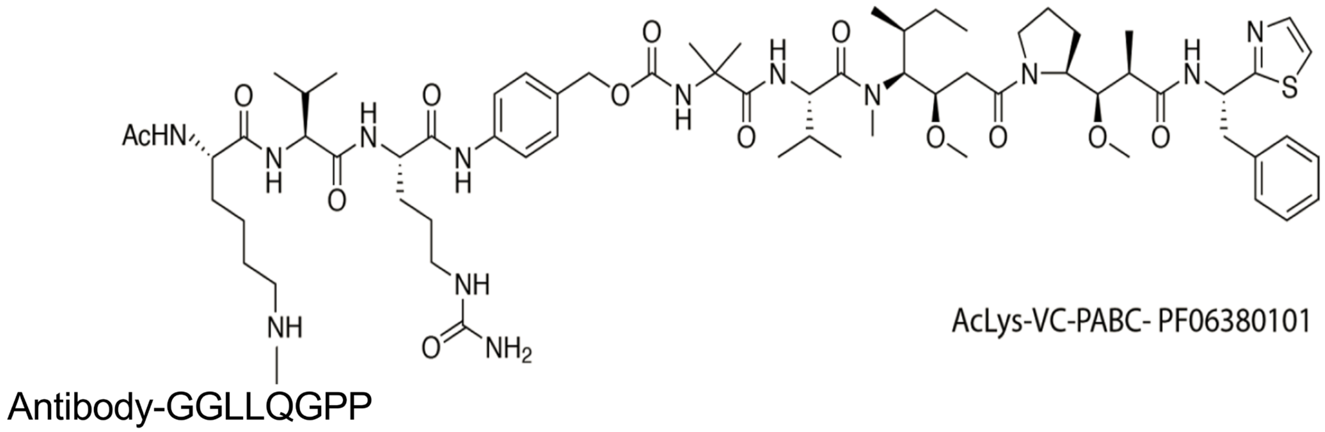

Supplementary Figure 1: Structure of RN765C. The chemical structure of the RN765C linker payload including the engineered transglutaminase tag GGLLQGPP which was added to the C-terminus of the light-chain constant domain.

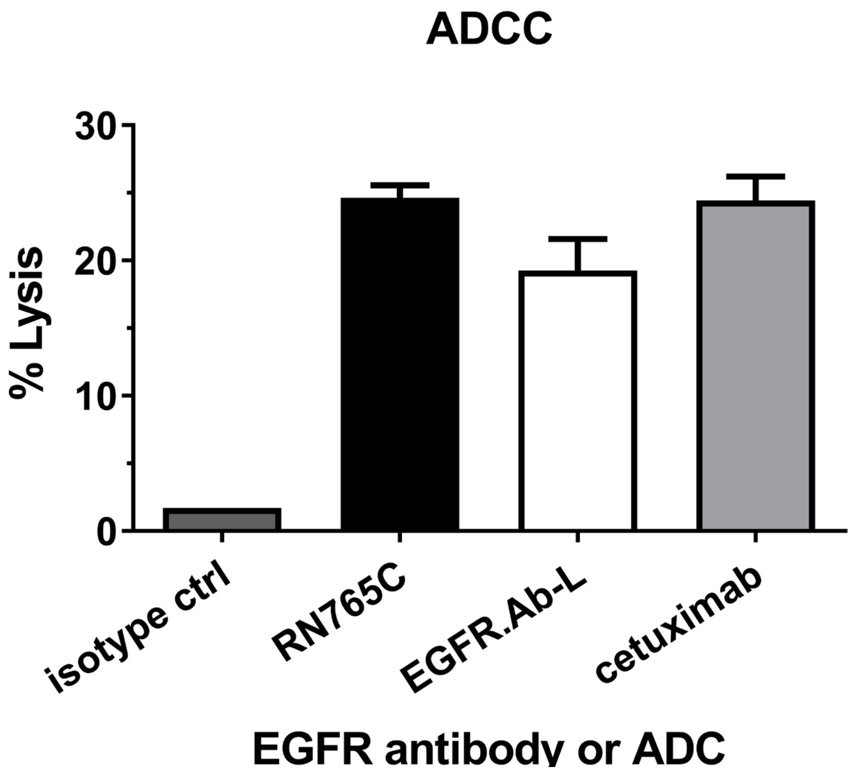

Supplementary Figure 2: RN765C retains robust antibody dependent cell cytotoxicity (ADCC). Fadu cells were exposed to purified CD56+ NK cells at an E:T ratio of 10:1 in the presence of the indicated antibodies or RN765C at 20 µg/mL for 4 h. Target cell lysis was measured by LDH (lactate dehydrogenase) release. All treatments were done in duplicate and wells with target cells alone or target cells plus NK cells were included as controls.
